# Retinoids: Mechanisms of Action in Neuronal Cell Fate Acquisition

**DOI:** 10.3390/life13122279

**Published:** 2023-11-29

**Authors:** Aysis Maria Koshy, Marco Antonio Mendoza-Parra

**Affiliations:** UMR 8030 Génomique Métabolique, Genoscope, Institut François Jacob, CEA, CNRS, University of Evry-val-d’Essonne, University Paris-Saclay, 91057 Évry, France; aysiskoshy@gmail.com

**Keywords:** stem cells, neuronal differentiation, retinoids, cell specialization

## Abstract

Neuronal differentiation has been shown to be directed by retinoid action during embryo development and has been exploited in various in vitro cell differentiation systems. In this review, we summarize the role of retinoids through the activation of their specific retinoic acid nuclear receptors during embryo development and also in a variety of in vitro strategies for neuronal differentiation, including recent efforts in driving cell specialization towards a range of neuronal subtypes and glial cells. Finally, we highlight the role of retinoic acid in recent protocols recapitulating nervous tissue complexity (cerebral organoids). Overall, we expect that this effort might pave the way for exploring the usage of specific synthetic retinoids for directing complex nervous tissue differentiation.

## 1. Introduction

Retinoic acid (RA) is a metabolic product of Vitamin A, not inherently produced by the mammalian body and thus ingested from external sources in the form of carotenoids and retinyl esters from plants and animals, respectively. Vitamin A was first discovered in 1913 [1], and numerous studies have demonstrated the necessity of this factor in early development, particularly in the development of the brain and vision [2,3]. The identification of the chemical structure can be attributed to Paul Karrer (1937, Nobel Prize in Chemistry) while the discovery of All-trans-Retinal as a crucial component in rhodopsin and thus subsequently necessary for vision is credited to George Wald (1967, Nobel Prize in Physiology and Medicine) [4]. Vitamin A, taken up by the body, is stored in the liver primarily but can also be found in extrahepatic sites such as the lungs, bone marrow, and kidneys [5].

## 2. Retinoic Acid Uptake and Metabolism

RBP4 (retinol-binding protein 4) is a protein found in the plasma capable of binding retinol and transporting it through the bloodstream to target cells containing the signaling receptor and transporter of retinol (STRA6) receptor [6]. Once retinol enters the cytoplasm, it binds RBP1 (retinol-binding protein 1), to be stored before its transformation into All-trans retinoic acid (AtRA).

Retinol is converted to retinaldehyde via alcohol dehydrogenases (ADH) and retinol dehydrogenases (RDH), and then to retinoic acid via retinaldehyde dehydrogenases (RALDH) [7]. The ADH family of enzymes can be ubiquitously expressed in embryonic and adult tissue (ADH5) or restricted to certain tissues (ADH1, ADH7) [8]. Knockout studies suggest that ADH enzymes are most likely involved in curbing retinol toxicity than participating in retinoic acid synthesis [9,10]. RDH enzymes, on the other hand, have a more pronounced effect in optical structures [11]. The RALDH family of enzymes consists of three enzymes—RALDH1, RALDH2, and RALDH3. Their distribution is site-specific and the availability of these enzymes serves as the rate-limiting step in retinoic acid synthesis. RALDH2, the earliest one to be expressed, first found in the primitive streak and mesodermal cells, is then localized to the somitic and lateral mesoderm, posterior heart tube, and rostral forebrain [12]. It is responsible for retinoic acid availability up to ~E8.5, after which RALDH1 and RALDH3 contribute to retinoic acid synthesis in the eyes and the olfactory system.

Retinoic acid resembles fatty acids due to the presence of a beta-ionone ring, a polyunsaturated side chain, and a polar end group [13], allowing for it to enter the cell easily through the plasma membrane. Now, in either an autocrine or paracrine fashion, retinoic acid diffuses into the neighboring cells, binds cellular retinoic acid-binding protein II (CRABPII), and moves into the nucleus. This is the point at which retinoic acid can now bind the retinoic acid receptors already bound to the DNA. After this action, retinoic acid moves out of the nucleus and is degraded by the CYP26 class of the cytochrome P450 (CYP450) superfamily of enzymes.

## 3. Retinoic Acid Receptors

Retinoic acid binds Retinoic Acid Receptor/Retinoid X Receptor (RAR/RXR) heterodimers, which are usually bound to retinoic acid response elements (RAREs) or retinoic X response elements (RXREs) in the promoter or enhancer regions of target genes [14]. RAREs usually consist of two direct repeats of either the hexameric sequence (A/G)G(G/T)TCA or the more relaxed motif (A/G)G(G/T)(G/T)(G/T)(G/C)A, separated by 1, 2, or 5 bp (DR1, DR2, and DR5 RAREs, respectively) [15]. This constitutive binding allows for the recruitment of corepressor complexes maintaining target gene repression.

The RAR and RXR receptors each exist as isotypes, RARα/β/γ and RXRα/β/γ, encoded by different genes, and each of them has its own isoforms, issued from alternative splicing and/or alternative promoter usage. While RXR receptors are only able to bind 9-cis retinoic acid (9-cis RA), RAR receptors can bind with high affinity AtRA and 9-cis RA. Each isotype receptor can be activated with specific ligands, that can be synthetic or naturally occurring. Natural agonists for RXR receptors are Docosahexaenoic acid, Lithocholic acid, and Methoprene acid [16,17]. There is an extensive list of pan-RXR synthetic agonists, including Bexarotene, LG100268, AGN194204, LG 101506, HX 630, and SR 11237. Phytanic acid, Honokiol, Peretinoin, AM-6-36, and CD3254 are RXRα specific agonists, while Danthron, derived from Chinese rhubarb, inversely is an RXRα specific antagonist [18]. Conversely, for RAR receptors, several synthetic ligands have been designed; for instance, Am 580, Am 80, BMS614, BMS753, AGN 193835, and AGN 193836 are RARα-specific. BMS641 and 2-thienyl-substituted dihydronaphthalene retinoids are modified ligands that are RARβ-specific, while BMS961 and α-hydroxyacetamide-linked retinoids have been described as potent for RARγ activity, and TTNPB as a pan-RAR agonist [17,19].

Interestingly, the capacity of each of the synthetic RAR agonists to regulate a specific transcriptional response has been described early on by Pierre Chambon’s team [20,21,22], and more recently, we have disentangled such a specific response at the level of the various controlled gene programs [23,24,25].

## 4. The Role and Distribution of Retinoic Acid in Embryogenesis

The distribution of the RARs in embryos differs from region to region. Numerous studies looked into the expression patterns of the RAR and RXR receptors, especially in early mouse embryogenesis [26,27,28], while a fair few also made comparisons with zebrafish [29,30,31] and xenopus models [32,33,34,35]. In these studies, the presence of these receptors was systematically addressed by taking into consideration the developmental stages described from a chronological point of view (e.g., pregastrulation, gastrulation, neurula, and organogenesis [36]).

Notably, while pregastrulation in mice and rat models does not reveal detectable levels of RAR/RXR gene expression, at gastrulation (E7.5), RARα and RARγ expression is ubiquitous and diffuse, and RARβ expression was found mainly in the lateral regions of the embryo [28]. Similarly, RXR expression during pregastrulation is also not defined, with RAR and RXR expression often overlapping and omnipresent throughout the embryo.

In addition to the presence of RAR/RXR receptors, the driving differentiation force for site-specific cell specialization leading to development structures depends on the bioavailability of RA. Retinoic acid is first detected at the primitive streak phase, i.e., E7.5 of embryonic development in mice, confirmed by the expression of class IV ADH mRNA seen initially in the posterior region along the primitive streak [8,12]. It is then found to be localized close to the trunk, hindbrain, and optic vesicle by E8.5–E9.5 [8,37]. 

**Figure 1:**
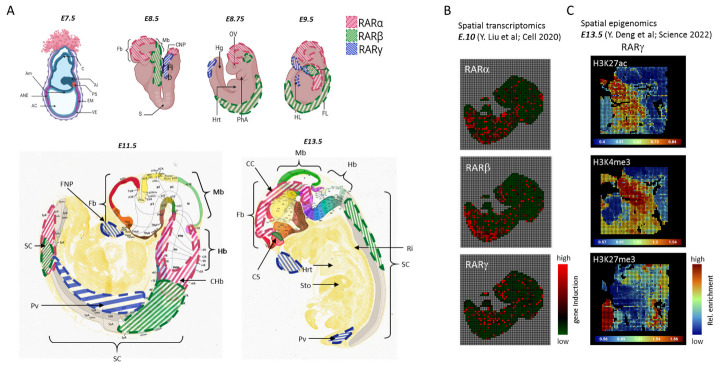
**CNS-relevant RAR isotype transcript expression in mouse embryogenesis.** (**A**) Schematic representation of the expression of RARα (red line shading), RARβ (green line shading), and RARγ (blue line shading) transcripts at different stages of mouse embryogenesis from E7.5 to E13.5, particularly in relation to CNS development. At E7.5, very low to undetectable levels of RAR expression are observed. RARα is first seen at E8.5, lowly expressed in the neuroectoderm, near to the caudal neuropore (CNP). By E11.5, it is more present in the posterior region of the spinal cord (SC) and in the caudal hindbrain (CHb), before being expressed primarily in the corpus striatum (CS) and corpus callosum (CC) at E13.5. RARβ (green line shading) is present mostly in the neural tube between E8.5 and E9.5, with a more localized presence at the caudal region of the spinal cord (SC) at E11.5 onwards, while RARγ (blue line shading) is first seen in the regressing primitive streak (PS; at E8.5), and it is undetectable later on in the brain and spinal cord (SC) neuroepithelium. Its expression is limited to the frontonasal process (FNP) and branchial arches (limb region; HL, FL). Top panel created on Biorender.com. Bottom panel (E11.5, E13.5) images taken from Allen Developing Mouse Brain Reference Atlases (https://developingmouse.brain-map.org/static/atlas) and overlayed with red/green/blue line shading to indicate RARα/β/γ expression, respectively. References made from studies [8,28,37,38,39,40,41]. AC: Amniotic cavity, Al: Allantois, Am: Amnion, ANE: Anterior neuroectoderm, C: Chorion, CC: Corpus callosum, CHb: Caudal hindbrain, CNP: Caudal neuropore, CS: Corpus striatum, EM: Embryonic mesoderm, Fb: Forebrain, FL: Forelimb, FNP: Frontonasal process, Hb: Hindbrain, Hf: Headfold, Hg: Hindgut, HL: Hindlimb, Hrt: Heart, Mb: Midbrain, OV: Optic vesicle, PhA: Pharyngeal arch, PS: Primitive streak, Pv: Prevertebrae, Ri: Ribs, S: Somites, Sto: Stomach, VE: Visceral endoderm. (**B**) RAR isotype over-expression revealed in spatial transcriptomics assay assessed in a *E.10* mouse embryo. The original data were produced by Liu et al. [42], and the visualization was performed within MULTILAYER [43,44]. (**C**) Histone modifications enrichment assessed within the gene coding region for the RARγ isotype, revealed by a spatial epigenomics assay performed on a E13.5 mouse embryo. Notice the coincident enrichment of the active marks H3K27ac and H3K4me3 within the frontonasal process region, as well as the enrichment of the repressive mark H3K27me3 in the developing CNS. The original data were produced by Deng et al. [45], and displayed in AtlasXplore [46].

Postgastrulation is characterized by the regional patterning of the neurectoderm, the formation of the somites, the migration of cranial neural crest cells, and more. During this span of time, RAR expression becomes more distinctive. As summarized in Figure 1A, by E8.5 to E13.5, there is an increased presence of RARα in the neuroectodermal region and in the regions distal to the caudal neuropore (CNP), as well as certain regions of the hindbrain up to rhombomere 6–7 (red line shading, Figure 1A). RARβ is highly expressed in early stage midbrain at E8.5, specific to the neural tube and the rostral regions of the mesodermal tissue, and then, later on, in the spinal cord [38,39,40] (green line shading; Figure 1A). RARγ transcripts, on the other hand, occupy the opposite end, being found mostly in the regressing primitive streak and in the tail region of the somite-axis [28], and almost completely absent from mesodermal tissue, as illustrated in the schematic representation provided in Figure 1A (blue line shading). 

By E13.5, RARα is majorly expressed in the corpus callosum and corpus striatum. At no point is RARγ (blue line shading) detected in the developing central nervous system (CNS). Its expression seems to be strongly repressed in this tissue layer, only appearing at the regions of frontonasal process (FNP), limb bud regions (HL, FL), and prevertebrae (Pv) from E9.5 onwards [40]. This summarized view concerning the distribution of RARs during embryo development is further supported with recent high-throughput data obtained by spatial technologies, like spatial transcriptomics or spatial epigenomics. These methods allow researchers to capture localized gene expression or histone modification enrichment signatures within a tissue section. Specifically, the team of Dr. Rong Fan at Yale University developed a microfluidics system allowing to transport molecular biology reagents in a grid-based manner on top of a tissue section, thus allowing distinct molecular barcodes to be ligated to either cDNA or chromatin cleaved regions associated with specific histone modification marks [42,45]. This methodology has been evaluated on mouse embryos, which allowed us to query for molecular signatures associated with RAR isotypes. Indeed, the preferential RARβ expression along the spinal cord is fully recapitulated in spatial transcriptomics assays (Figure 1B: E.10; Y. Liu et al. [42]), and the described repression of RARγ in the developing CNS is fully confirmed by spatial epigenomics profiling targeting the histone modification H3K27me3, the most well-characterized gene silencing marker [47]. Similarly, the RARγ preferential expression at the frontonasal process (FNP), displayed in Figure 1A, correlates with the presence of the active marks H3K4me3 and H3K27ac, which concomitantly are known to create an open chromatin environment permissible to transcription [47] (Figure 1C: E13.5; Deng et al. [45]). 

Retinoic acid functionality also works in synchrony with complementary signals such as fibroblast growth factor (FGF-8), sonic hedgehog (SHH), and bone morphogenetic proteins (BMPs). Retinoic acid plays a role primarily in the growth and differentiation of the posterior structures, while in the anterior part of the embryo, its activity is regulated by the RA-degrading CYP450 enzymes CYP26A1 and CYP26C1, promoting the anterior-posterior patterning [48,49,50]. Retinoic acid patterns the anteroposterior axis by being synthesized in the posterior mesoderm. The presence of CYP26C1 in the anterior mesoderm prevents the influence of retinoic acid in these regions, leading to the emergence of the hindbrain from the neural plate. In the dorsoventral axis of the developing neural tube, retinoic acid is produced at the somite sites, along with SHH and BMP expressed ventrally and dorsally, respectively. FGF-8 expression is detectable at the posterior end of the extending neural tube, and these factor gradients predict the ultimate fate of specialized neurons that emerge such as interneurons, sensory neurons, and motor neurons [51,52,53].

## 5. Neuronal Cell Specialization Studies in Animal Models

Beyond its major role in patterning, retinoic acid also plays a role in neuronal differentiation. In addition to its direct action in the regional patterning of the neurectoderm, there have been several studies that have discussed the importance of retinoic acid for the generation of motor neurons and the ventral progenitors [51,54,55], subsets of GABAergic or dopaminergic neurons [56,57,58], as well as terminal neuronal differentiation in ventricular and subventricular zones [59].

Among these studies, the role of retinoic acid in the emergence of motor neurons during the development of the CNS in chick embryos was already studied at the end of the 1990s by Shanthini Sockanathan and colleagues [54]. 

Specifically, during CNS development, some proliferating stem cells either remain undifferentiated or they can leave the cell cycle to become progenitors and eventually terminally differentiated cell types like neurons or glia, in an inside-out fashion [60]. This means that the earlier born neurons are found inside while the late-born neurons feature more towards the apical side. In a similar way, medial lateral motor column (LMC) neurons, that project to ventral limb muscles, leave the cell cycle first and form the first layers, followed by lateral LMC neurons, projecting to dorsal limb muscles, that move past the medial LMC neurons to their endpoint. Evidence proved the effect of retinoids synthesized by neurons that influence the differentiation capacity of the lateral LMC neurons, but also the quantity, subtype identity, and timing of maturation at the level of the limbs. The longitudinal limb development, shown to be defined by a proximal source of retinoic acid and a distal source of FGFs, is concomitant with the motor neuron innervation process issued from the LMC, driven by the patterned expression of nearly two dozen individual Hox genes and the conserved cofactor forkhead box P1, which together control retinoic acid synthesis in limb-innervating motor neurons [61].

The combined and harmonious signaling of retinoic acid, but also FGF-8 and SHH, is vital in inducing the motor neuron identity as well as patterning of the subsequent ventral neural structures. For example, oligodendrocyte transcription factor 2 (Olig2) expression—driven by retinoic acid and SHH action—marks the identity of motor neuron progenitors and is tightly linked to the expression of homeobox domain (HD) regulators. The expression of Olig2 is negatively regulated by the expression of NK2 homeobox 2 (Nkx2.2) (driven by SHH), while Olig2 negatively regulates the expression of the HD factor Pax6 [62]. 

Additionally, retinoic acid has been discovered to have a dose-dependent effect on the identities of differentiating p3 V3 neurons versus serotonergic neurons [58,63]. The p3 progenitors are the most ventral progenitor domain present in the spinal cord and hindbrain, both being equivalent populations. They give rise to the glutamatergic V3 interneurons in the spinal cord and serotonergic (5-HT) neurons in the hindbrain. Conclusively, these studies were able to show that higher levels of retinoic acid are present in the p3 [V3] domain, and via the retinoid acid receptor signaling, one of the downstream targets, Notch, has a direct role to play in the activity of achaete-scute homolog 1 (Ascl1). Lower levels of Ascl1 confirm a glutamatergic V3 interneuron fate from p3 progenitors, and the opposite is true for serotonergic fate commitment in the hindbrain. 

In the zebrafish embryo, particularly in the posterior hindbrain, the medulla and the area postrema, noradrenergic (NA) neuronal differentiation is initiated by the action of retinoic acid [56]. Similarly, FGF-8 is the signal inducing noradrenergic specification in the locus coeruleus. In both cases, precursor neurons require the expression of transcription factor AP-2α, in order to terminally mature into NA neurons. 

Retinoic acid is also responsible for GABAergic differentiation in mouse embryos at E14.5 in the subventricular zone of the basal ganglia, due to the expression of RALDH3 [57]. This is also seen in the lateral ganglionic eminence (LGE) where the same pathway leads to endogenous retinoic acid, required for GABAergic neuronal differentiation. By E18.5, the role of retinoic acid is to maintain GABAergic differentiation in the LGE, primarily through the stimulation of 67 kDa glutamic acid decarboxylase (GAD-67) activity. But it is not yet clear which RAR/RXR receptors might be linked to this activity, since Gad67 does not show evidence of a canonical RARE element in its promoter region. This being said, RARβ seems to be necessary for the development of striatonigral projection neurons. Indeed, studies in RARβ-ablated mouse embryos showed reduced levels of 65 kDa glutamic acid decarboxylase (GAD-65), dopamine D1 receptor, and substance P found in these neurons, at E16.5 [59]. At E18.5, there is a partial recovery of Gad67 expression, suggesting neurogenesis of some GABAergic neurons. This is also confirmed by the lower levels of Ascl1, a marker for neural progenitors and a GABAergic neuron determinant [64], concurrently with increased homeobox *Meis* gene expression, a marker for postmitotic neurons, seen in the ventricular and subventricular zones. This marked reduction in proliferation and substitution with premature maturation can be explained by the activity of FGF-3, a direct target of retinoid receptors [65], which, at E13.5, is the main FGF functioning in these zones. This interplay between retinoic acid and FGF-3 holds the key in balancing the populations and chronological events from neural progenitors and their proliferation to terminal neuronal differentiation.

## 6. In Vitro Studies Modeling Neuronal Cell Specialization

Modeling neuronal differentiation in vitro, driven by retinoic acid action, was initially made possible with the help of the P19 embryonic carcinoma cells. This cell line was first derived by McBurney and Rogers in 1982, from the transplantation of a 7.5-day-old embryo onto the testis of a mouse. The resulting teratocarcinoma could be cultured in vitro, growing rapidly without the need for irradiated mouse feeder cells [66]. They do not form tumors unless injected into neonates and provide multiple advantages for their use in studying cell differentiation early on. Their advantages lie in their ease of culture, multipotency, anchorage-independence, and lack of contact inhibition [67]. This was beneficial to study the influence of externally introduced chemicals on a simplified in vitro neuronal development model.

A number of studies have looked into the effects of molecules such as dimethyl sulfoxide (DMSO) and retinoic acid, in their abilities to induce any of the 3 germ layers [68]. DMSO treatment on a given clone of P19 cell aggregates pushes the cells to differentiate into cardiac and muscle-like tissue. DMSO exposure at 0.5–1% has been shown to cause the outer cell layers of the P19 embryoid to mature into cardiac, skeletal, and epithelial cells, mostly expressing smooth muscle α-actin [68,69,70]. Within 6 days, striated cardiac tissue is evident, exhibiting contractile movements. By 9–10 days of differentiation, skeletal tissue appears. Additionally, these developments are seen only in embryoid bodies and not when the cells have been seeded as a monolayer culture prior to DMSO treatment.

What DMSO is to cardiac tissue, retinoic acid is to neurons. The treatment of retinoic acid on P19 aggregates at concentrations of 1 µM is the ideal method of generating neurons within a 10-day span [71]. 

In an in vitro system, allowing to induce neuronal cell differentiation by the use of a single compound, i.e., retinoic acid, provides a suitable method to elucidate the role of the various RAR/RXR nuclear receptors during this process. Importantly, the capacity of driving neuronal differentiation by the sole action of the RARα agonist BMS753, and not by the activation of the other two RAR receptors, was initially described by Chambon’s team in the 1990s [21]. This major observation was further encountered in our previous work based on the use of synthetic RAR agonists for inducing differentiation in both P19 cells (leading to neuronal differentiation) and F9 embryonic carcinoma cells (leading to endodermal differentiation) [24,72,73]. This detailed study allowed to identify RAR-direct targets during the first 48 h of differentiation, as well as their downstream regulatory targets leading to either neurogenesis or endodermal differentiation, but also to identify a common gene program.

**Figure 2:**
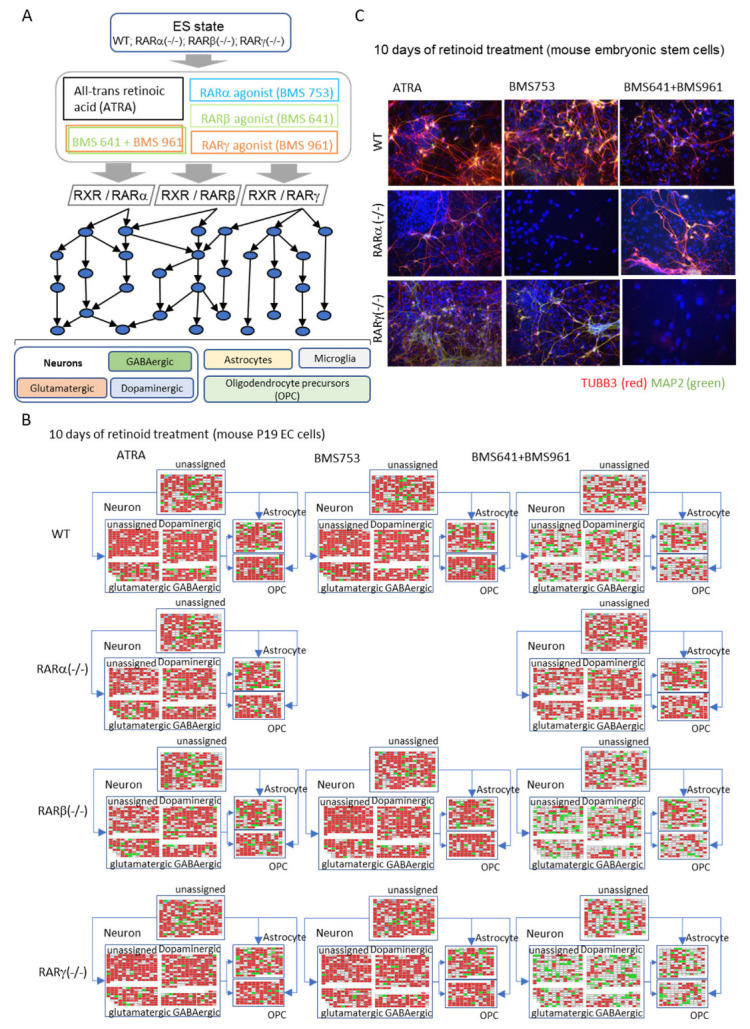
**Disentangling neuronal cell specialization driven by retinoid action.** (**A**) Scheme illustrating the strategy applied by Koshy et al. [25] to discern the role of each RAR isotype receptor during cell specialization. Wild-type (WT) and RAR knockout P19 mouse embryonal carcinoma stem cells (ES state) are either treated with the pan-RAR agonist (AtRA), or with each of the indicated synthetic agonists activating specific RAR isotypes, as well as the combination of the RARγ (BMS961) and RARβ (BMS641) agonists. The use of these RAR agonists is expected to initiate a transcription regulation cascade, leading to the induction of gene programs defining the emergence of the various neuronal subtypes as well as glial cells. (**B**) Gene regulatory networks established from transcriptomes assessed in P19 embryonal carcinoma cells (WT or RAR knockout lines) treated with each of the indicated RAR agonists (10 days). Notice that the concomitant activation of both RARβ and RARγ receptors leads to the activation of genes associated with the indicated neuronal subtypes (dopaminergic, glutamatergic, and GABAergic) as well as with astrocytes and oligodendrocyte precursors (OPC) in WT and RARα (−/−) cells. Data corresponding to RARα (−/−) cells treated with the BMS753 ligand were not assessed since cells do not differentiate in this context. (**C**) Immunostaining micrographs revealing the presence of β-III tubulin positive neuronal precursors, as well as mature neurons (MAP2) in mouse embryonic stem cells differentiated in the presence of either the pan-agonist AtRA, the RARα agonist BMS753, or the combination of the BMS641 and BMS961 agonists, activating the RARβ and RARγ receptors. This synergistic action of the BMS641 and BMS961 agonists has been also confirmed in Rarα(−/−), but not Rargγ(−/−) knock-out cells [25].

More recently, we found that, while RARβ or RARγ agonists are not able to induce neurogenesis, their synergistic action surprisingly allows them to do so, demonstrating that there exist redundant pathways driven by each RAR subtype, but also their distinctive influence [25]. In this study, in addition to the use of synthetic RAR agonists, we generated P19 CRISPR-Cas9 RAR knock-out lines, providing means to evaluate the redundancy between RARs (Figure 2A). Specifically, what was relevant was the ability of RARβ and RARγ coactivation to lead to a rescued neuronal phenotype at the end of 10 days of cell differentiation in P19 embryonic carcinoma cells, while the individual activation of these receptors with their synthetic agonists (BMS641 and BMS961, respectively) did not do so. Taking a closer look at the global transcriptional profiles, within reconstructed gene regulatory networks, revealed that 45–60% of upregulated genes associated with markers for neurons, astrocytes, and oligodendrocyte precursor cells were recovered from the RARβ and RARγ coactivation in wildtype cells, but more than 70% recovery was seen with the same markers from RARβ and RARγ coactivation in RARα (−/−) cells [25]. In contrast, RARβ and RARγ coactivation in either RARβ (−/−) or RARγ (−/−) cells presented a poor yield of upregulated genes associated with the studied neuronal subtypes or glial cells (Figure 2B), in agreement with the low cell differentiation performance. Finally, it is worth mentioning that these findings performed in P19 embryonal carcinoma cells were also confirmed in mouse embryonic stem cells, confirming the potency of the synergistic action of both RARβ and RARγ agonists to lead to neuronal cell specialization (Figure 2C) [25].

Similar to our study, Podleśny-Drabiniok and colleagues highlighted not just the role of retinoic acid in driving neurogenesis but also the additional layer of regulation by the retinoid receptors that leads to cell specialization [74]. Using individual synthetic RAR agonists allowed them to detect the neural cell identity achieved in P19 embryoid bodies over a period of 10 days. On exposure to AtRA, 88.5% of the cell population expressed β-III tubulin (a pan-neuronal marker), and 90% of these cells over-expressed markers associated with GABAergic neurons (Gad65/67). The authors also used synthetic agonists targeting specific RAR isotypes. Notably, in contrast to our findings, cells treated with a RARγ agonist (CD666) were most successful in generating GABAergic neurons, with a 77% yield, while those treated with RARβ agonist (BMS641) were the least efficient, with only a 28% yield. Interestingly, a subpopulation of dopaminergic neurons was also confirmed by the presence of the dopamine transporter (DAT) and the absence of the noradrenaline transporter (NET), and all of them were GABAergic.

While both studies provide evidence for the role of individual receptors distinctly influencing terminal cell fate [25,74], there are several key differences which might explain the discordant findings. For instance, different RARγ selective agonists were used in these studies (BMS961 versus CD666). This is relevant since Podleśny-Drabiniok et al. observed no differences in cell differentiation marker expression when using only RARγ versus RARβ and RARγ agonists. The potential of a cross-activation of the RARβ program by the agonist CD666 remains plausible, as discussed by Million et al. [75]. Furthermore, the experimental setup used in our study follows monolayer differentiation conditions, while Podleśny-Drabiniok et al. differentiated P19 embryonic carcinoma cells in embryoid bodies. Thus, the main takeaway message from our study is the synergistic activity of RARγ and RARβ in recovering the neuronal differentiation potential that is not possible when each of these receptors are activated individually, while in the case of Podleśny-Drabiniok et al., RARγ activation alone can lead to a striatopallidal-like neuronal population.

Recent studies are further exploiting the capacity of retinoic acid independently and in combination with other growth factors and morphogens, by mimicking embryogenesis in vitro. Indeed, as discussed before, retinoic acid, released from the somites and the SHH gradient produced by the floor plate and notochord [76,77,78], provides a rostral-caudal and ventral-dorsal gradient, respectively, influencing the emergence of the ventral progenitor interneuron domains (p0–p3) and a progenitor motor neuron domain (pMN) arranged in the ventral-dorsal axis, which ultimately mature into the ventral interneuron classes and motor neurons [76,79,80].

V2a interneurons, found in the spinal cord and the respiratory centers of the hindbrain, are synthesized from mouse embryonic stem cells that are treated with low concentrations of retinoic acid and high concentrations of SHH agonist (Pur) [81]. Additionally, in order to specifically choose the commitment of these cells for V2a interneuron production over V2b interneurons, Notch1 signaling is inhibited. This also has an auxiliary benefit in preventing glial cell proliferation. A more dorsal phenotype of neural differentiation can be achieved by switching to higher concentrations of retinoic acid and their positional identity is determined within 48 h of exposure [78].

Motor neuron generation is frequently researched and almost always involves the introduction of the morphogens retinoic acid and SHH, with retinoic acid inducing a caudalizing effect (especially noticeable in the embryoid bodies of neural progenitor cells), and SHH creating a motor neuron progenitor specialization [82].

Conversely, the generation of cerebellar neurons requires a host of signaling molecules at particular timepoints, namely, the cerebellar organizers FGF-8 and retinoic acid, and subsequent treatment with the dorsalizing molecules WNT, BMP6/7, and growth differentiation factor 7, followed by SHH and jagged1 protein, to induce the proliferation of granule cell progenitors (GCPs) along with medium cultured by cerebellar glial cells [83].

These same studies extended into the use of human pluripotent stem cells (hPSC) as a research model, paving the way for more clinically relevant and transferable knowledge on neuronal cell fate.

In the case of motor neuron generation, retinoic acid and SHH treatment are deemed necessary for Sox1+ neuroectodermal cells derived from human embryonic stem cells (hESC), preferably at low concentrations, with retinoic acid treatment occurring early on [84]. This is to avoid regional specificity that the cells acquire once they become Pax6+/Sox1+. Their effects can be seen in monolayer cultures at 2 weeks, with extensive neurite outgrowth and the differentiated neurons having a positive profile for Islet1/2, and 50% of those being HB9+. These double positive marker cells confirm the generation of spinal motor neurons in the cell culture. As mentioned before, retinoic acid here is acting as the caudalizing factor while Shh is the ventralizing inducer. The role of retinoic acid is seen in its effect on the upregulation of Shh as well as Class II HD profile proteins (Olig2, Nkx2.2, and Nkx6.1), essential for motor neuron specification. This protocol received an update in 2013, with the addition of SAG (smoothened agonist) [85]. It generates a higher yield of motor neurons in a shorter time frame, of which there is a varying expression pattern of homeodomain protein Hb9 and Islet1, mimicking in vivo distribution patterns.

The NTERA2 clone D1 (NT2/D1) is a human embryonic carcinoma cell line that has been frequently researched to study cell fate and differentiation in response to retinoic acid. These cells can be cultured in monolayer or spheroid cultures in the presence of retinoic acid to give rise to cells with a neural signature [86]. Furthermore, Coyle and colleagues demonstrated the capacity of this cell line to produce a range of mature neurons (GABAergic, glutamatergic, dopaminergic, and cholinergic) [87], while a previous study by Zeller and Strauss explored the capacity of NT2/D1 cells to differentiate into cholinergic neurons [88]. A further study performed the expression profile of RAR isotypes during NT2D1 differentiation, demonstrating that RARα is induced within a day of the addition of retinoic acid, RARβ remains at a constant expression, while RARγ expression decreases overtime [89]. However, in our literature survey, we were not able to find studies linking RAR isotype action to directing cell fate acquisition in this model system.

Neuronal differentiation using retinoic acid continues to be tested to determine the best possible conditions for generating neurons. Longer exposure times to retinoic acid, culturing cells as embryoid bodies especially during their exposure to retinoic acid, and culturing cells at higher cell densities are factors shown to increase neuronal yield [90]. Higher cell densities promote cell crosstalk and have an effect on the upregulation of neurogenesis factors like Sox2, neurogenic differentiation factor 1, and Pax6, ultimately influencing the formation of neuron-specific β-III tubulin-positive cells.

Many of these studies provide protocols to generate a subset of neural identities from a starting embryonic stem cell culture with varying percentages, yield, and heterogeneity, and heavily dependent on the concentrations of morphogens, growth factors, and culture conditions. Nevertheless, the link between specific RAR receptors and their ultimate cell fate influence still remains to be decoded, as illustrated by Podleśny-Drabiniok and colleagues [74], as well as by our recent study [25].

## 7. Perspectives

Preliminary studies often rely on in vitro monolayer cultures owing to their simplicity and ease of deducing cause and effect. However, we are now approaching more complex and integrated model systems to study tissue development and disease. For example, retinoic acid is clearly demonstrated to be a crucial element for brain organoid development [91,92]. At the early stages of development, it directs cells to commit to the neuroectodermal pathway, whether it be in the case of unpatterned whole brain organoid generation or specifically for cerebral or dorsally patterned brain organoids. The current state of research is moving quickly to include single cell analysis [93,94,95], spatial transcriptomics, and lineage tracing [96,97,98], and we are already witnessing the possibility of growing embryo-like structures from stem cells (stembryos) [99,100]. Thus, the need to study the influence of each RAR isotype in brain development and neural cell fate remains quite strong, possibly providing a means for targeted therapy in the near future.
